# The use of ‘PICO for synthesis’ and methods for synthesis without meta-analysis: protocol for a survey of current practice in systematic reviews of health interventions

**DOI:** 10.12688/f1000research.24469.2

**Published:** 2021-01-29

**Authors:** Miranda S. Cumpston, Joanne E. McKenzie, James Thomas, Sue E. Brennan

**Affiliations:** 1 School of Public Health and Preventive Medicine, Monash University, Melbourne, VIC, 3004, Australia; 2 EPPI-Centre, UCL Social Research Institute, University College London, London, WC1H 0NR, UK

**Keywords:** Systematic reviews, meta-analysis, synthesis, subgroup analysis, narrative synthesis, synthesis without meta-analysis, PICO

## Abstract

**Introduction:** Systematic reviews involve synthesis of research to inform decision making by clinicians, consumers, policy makers and researchers. While guidance for synthesis often focuses on meta-analysis, synthesis begins with specifying the ’PICO for each synthesis’ (i.e. the criteria for deciding which populations, interventions, comparators and outcomes are eligible for each analysis). Synthesis may also involve the use of statistical methods other than meta-analysis (e.g. vote counting based on the direction of effect, presenting the range of effects, combining P values) augmented by visual display, tables and text-based summaries. This study examines these two aspects of synthesis.

**Objectives:** To identify and describe current practice in systematic reviews of health interventions in relation to: (i) approaches to grouping and definition of PICO characteristics for synthesis; and (ii) methods of summary and synthesis when meta-analysis is not used.

**Methods:** We will randomly sample 100 systematic reviews of the quantitative effects of public health and health systems interventions published in 2018 and indexed in the *Health Evidence and Health Systems Evidence* databases. Two authors will independently screen citations for eligibility. Two authors will confirm eligibility based on full text, then extract data for 20% of reviews on the specification and use of PICO for synthesis, and the presentation and synthesis methods used (e.g. statistical synthesis methods, tabulation, visual displays, structured summary). The remaining reviews will be confirmed as eligible and data extracted by a single author. We will use descriptive statistics to summarise the specification of methods and their use in practice. We will compare how clearly the PICO for synthesis is specified in reviews that primarily use meta-analysis and those that do not.

**Conclusion: **This study will provide an understanding of current practice in two important aspects of the synthesis process, enabling future research to test the feasibility and impact of different approaches.

## Introduction

**Figure 1.**
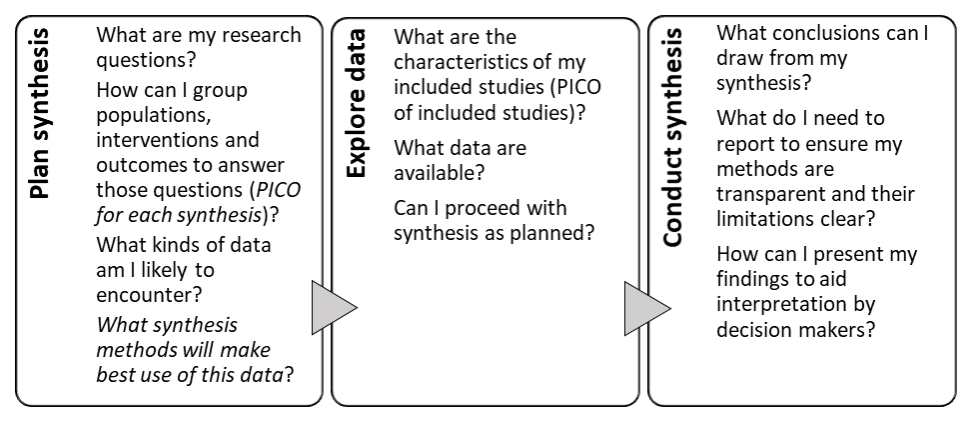
Steps in the evidence synthesis process. Steps in evidence synthesis are to plan synthesis, explore data and conduct synthesis. Key issues examined in this study identified in italics. PICO = Population, Intervention, Comparator, Outcome.

Systematic reviews provide a method for collating and synthesising research, and are used to inform decision making by clinicians, consumers, policy makers and researchers ^1^. In health intervention research, the synthesis component of systematic reviews is often narrowly considered as the use of statistical methods to combine the results of studies, primarily meta-analysis, and much of the available guidance focuses on this approach. However, ‘synthesis’ can be considered more broadly as a process, beginning with defining the review questions, planning the groups to be compared, examining the characteristics of the available studies and their data, and applying appropriate methods to present and synthesise quantitative data from among multiple options (see Figure 1). Decisions made early in the process have important impacts on the information included in the synthesis, and meta-analysis may not always be possible or appropriate.

In this study, we plan to examine two intertwined aspects of synthesis that commonly challenge authors of systematic reviews examining the effects of health interventions (identified in italics in Figure 1): approaches to planning how studies will be grouped for synthesis within the review (the ‘PICO (Population, Intervention, Comparator, Outcome) for each synthesis’); and the application of methods other than meta-analysis to summarise and synthesise quantitative results (hereafter described as ‘other synthesis methods’). There has been limited examination of the range of approaches used to define the PICO for each synthesis and which other synthesis methods are used in current practice. Yet, these are essential aspects of the synthesis in systematic reviews.

Recent guidance published in the *Cochrane Handbook for Systematic Reviews of Interventions* ^2– 4^ has outlined proposed methods for specifying the PICO for each synthesis and a range of other synthesis methods. Reporting guidance for ‘synthesis without meta-analysis’ (SWiM) has also been published ^5^, covering these topics. However, further research is required to understand current practice and investigate how review authors approach the PICO for each synthesis and other synthesis methods.

We now expand on the concept of ‘PICO for each synthesis’ and methods for synthesising and presenting findings other than meta-analysis.

### PICO for each synthesis

In reviews of the effects of interventions, authors commonly use the ‘PICO’ framework to prespecify the populations, interventions, comparators and outcomes that will be used to determine whether studies are eligible for the review ^6^. While this definition of the ‘PICO for the review’ is viewed as a core component of a systematic review, more specific criteria are likely to be needed to define which groups of studies will contribute to each analysis within a review: the ‘PICO for each synthesis’. The PICO for each synthesis can be considered an operationalisation of the review objectives.

The process for defining the PICO for each synthesis ideally involves identifying characteristics (e.g. of the intervention or population) that may be expected to modify the intervention effect; clearly labelling and defining groups based on these characteristics (these may be based on an existing classification system if available); and planning how these groups will be used in synthesis and reporting. Groups may be analysed together in an overall synthesis, or they may be considered in separate syntheses ^4^. Within an overall analysis, the defined groups may be used to explore any differences in the estimated effects (i.e. to explore statistical heterogeneity through the use of subgroup analysis). An example demonstrating the distinction between the PICO for the review and the PICO for each synthesis is presented in Box 1.

**Box 1. Example: PICO for the review and PICO for each synthesis**

In a review of psychosocial interventions for smoking cessation ^7^, the PICO for the review included *any* psychosocial intervention in pregnant women to help them stop smoking.

One of the objectives of the review was to examine “the effectiveness of the main psychosocial intervention strategies in supporting women to stop smoking in pregnancy (i.e. counselling, health education, feedback, social support, incentives, exercise)”. In order to meet this objective, a series of syntheses were presented within the review to assess the effects of each intervention strategy. So, for example, the PICO for the first synthesis presented included any counselling intervention for women during pregnancy compared to usual care, measuring the outcome of smoking abstinence in late pregnancy.

Another objective was to determine whether psychosocial interventions were effective in general. To address this objective, all intervention types were included in a single meta-analysis. Within this analysis, single, multi-component, and tailored interventions were presented as subgroups, to examine whether intervention effects were modified by having multiple or tailored components.

(Adapted from: Chapter 3, *Cochrane Handbook for Systematic Reviews of Interventions* ^4^. PICO = Population, Intervention, Comparison, Outcome.)

Providing such definition has important advantages. Creating a consistent language to describe different groups or interventions can increase clarity of terminology for readers, allowing authors to compare features between the included studies and make consistent, transparent decisions about grouping similar studies for inclusion in a synthesis ^3^. 

The PICO for synthesis also provides a framework for examining similarities and differences in the characteristics of studies contributing to each analysis, facilitating qualitative synthesis of characteristics needed to interpret results. This qualitative synthesis is a particularly import feature of reviews where there is diversity in study characteristics that may explain findings (e.g. intervention complexity, different study designs) ^8^. Such diversity sometimes triggers a decision not to use meta-analysis, and instead adopt alternative methods to synthesise and present findings. In these circumstances, it is common for authors to refer to their synthesis methods as ‘narrative’ ^9^, reflecting the integration of the synthesis of quantitative results from studies with the qualitative synthesis of study characteristics. In this study, we distinguish between these elements and, in the section that follows, focus on the methods used to combine quantitative data on intervention effects using a statistical technique and to present the results of these analyses.

### Synthesising and presenting findings without meta-analysis

Many systematic reviews examining the effects of health interventions use meta-analysis of effect estimates to combine the results of studies ^9, 10^. However, it is estimated that between 35% and 56% of systematic reviews do not use meta-analysis at all ^9, 10^, and a larger percentage of reviews do not use meta-analysis for at least some outcomes. The reasons for not undertaking meta-analysis vary, but a commonly reported reason is that the included studies do not report data that is amenable to meta-analysis ^9, 11^. For example, studies may report effect estimates without a measure of variance, or only report the direction of effect, or they may report different effect measures that cannot be transformed into a common effect measure ^2^. Diversity of study characteristics and the presence of statistical heterogeneity are other reasons given for not meta-analysing, but these are more contentious. The first brings into question whether any synthesis is appropriate, while the second may be addressed by using extensions to meta-analysis (e.g. meta-regression, prediction intervals) that attempt to explain or encompass heterogeneity ^2, 11^.

When meta-analysis of effect estimates is not possible, a range of summary and other synthesis methods are available (see examples in Table 1). These methods include alternative statistical synthesis methods, such as presenting summary statistics (e.g. range of effects), combining P values, and vote counting based on direction of effect. These synthesis methods may be augmented using tables, visual display (e.g. harvest plots, albatross plots) and, where synthesis is not appropriate, structured summaries of the results of individual studies ^2, 12^.

**Table 1.** Examples of data collection items.

Category	Examples of data collection items
Review characteristics	• Reference information • No. of included studies • PICO for the review • Availability of a protocol or registration (e.g. PROSPERO) • Methodological and risk of bias characteristics (quality scores by Health Evidence ^14^ or Health Systems Evidence ^15^, selected items from ROBIS tool ^16^)
PICO for each synthesis	For each PICO element plus study designs: • Groupings described in the review • Whether groupings were specified in enough detail for replication • Basis for grouping (classifications outlined in the *Cochrane Handbook for Systematic Reviews of* *Interventions ^4^:* • Population: e.g. intended recipient of intervention; disease/condition, participant characteristics (PROGRESS-Plus framework ^17^), setting • Intervention/Comparator: e.g. intervention characteristics (TIDieR framework ^18^); handling of inactive controls, multi-component interventions, co-interventions • Outcome: e.g. outcome domains, measurement tools/methods, time points • Study design: e.g. design, design features, certainty of the evidence (GRADE), risk of bias, study size • Roles in synthesis explicitly specified for groupings • Groupings used in practice in synthesis • Reporting of changes to planned groupings
Synthesis, summary and presentation methods	• Summary methods (approach to summarising individual study results in text and tables) • Synthesis methods (e.g. meta-analysis, descriptive statistics combining P values, vote counting based on statistical significance, vote counting based on the direction of effect) • Methods to investigate or encompass heterogeneity (e.g. subgroup analysis, meta- regression, prediction intervals, non-parametric methods) • Presentation methods (e.g. tables, forest plots, box-and-whisker plots, bubble plots, albatross plots, harvest plots, effect direction plots, stacked bar plots, funnel plots) • Methods used to select among multiple effect estimates eligible for a synthesis • Reporting of changes to planned methods

Examples of data items to be collected from sample, including systematic review characteristics, PICO for each synthesis and summary and synthesis methods. PICO = Population, Intervention, Comparator, Outcome. The complete draft data dictionary is available as *Extended data ^19^*

Other synthesis methods provide more limited information for health care decision making in comparison to meta-analysis (for example, providing information on the likely direction of effect, rather than an estimate of its magnitude ^2^). Nevertheless, structured summary or synthesis approaches may be preferable to simply presenting an unstructured description of study-level results, in which there is a risk that authors may privilege the results of some studies over others without appropriate justification, possibly introducing bias ^9^.

Importantly, the use of other synthesis methods may alter the nature of the question answered by the review and the type of reasoning used to reach conclusions ^2, 13^. 

### Research context

We are unaware of other studies that have explicitly examined approaches to defining the PICO for each synthesis and planning comparisons. One cross-sectional study collected data on which PICO characteristics (e.g. population) were used to group studies for presentation or analysis within systematic reviews ^9^. However, this study did not capture more detailed information on the basis of these groupings (e.g. was the population grouped by clinical disease characteristics, age or socioeconomic status), nor precisely how these groups were used in the synthesis.

Previous studies have examined the synthesis methods used in systematic reviews, and have estimated the percentage of reviews with and without meta-analysis ^10, 11, 20^. One study examined systematic reviews of public health interventions that did not use meta-analysis in further detail ^9^. They captured data on the use and reporting of “narrative” (text-based) synthesis and methods to investigate heterogeneity, but specific details of the synthesis methods used in the reviews were not captured. Another study examined the use of outcome groupings in synthesis and the use of methods other than meta-analysis, but the study was limited to Cochrane systematic reviews published before 2012 ^21^.

### Objectives

The objectives of this study are to identify and describe current practice in systematic reviews examining the quantitative effects of public health and health systems interventions in relation to:

1. Approaches to grouping and definition of PICO characteristics for synthesis.
2. Methods of summary and synthesis when meta-analysis is not used.

Here we report the proposed methods for a cross-sectional study of a sample of systematic reviews.

## Methods

### Overview

We will identify a sample of systematic reviews examining the quantitative effects of public health or health systems interventions. We will identify and describe the methods used to define the PICO for each synthesis and the methods used to summarise and synthesise results, including meta-analysis and other methods. Two authors will undertake study selection. One author will undertake data extraction, and a second author will conduct independent data extraction from a subset of studies. Any amendments or additions to this protocol will be reported in resulting publications.

### Eligibility criteria

We will include systematic reviews that meet the following criteria:

1. A study that aims to synthesise the results of primary studies, states eligibility criteria for inclusion of studies, and reports a search strategy to identify potentially eligible studies.
2. Examines quantitative effects of any public health or health systems intervention, including policies, programs and strategies, as well as treatments and elements of care.
3. Includes at least one comparison with at least two studies, where a comparison is defined as examining the effect on an outcome of an intervention compared with a specific alternative.
4. Published in English.

We will exclude systemic reviews that:

1. Synthesise the results of other systematic reviews, such as overviews of reviews.
2. Answer questions that are not about effectiveness, for example prevalence, association, unplanned environmental exposures, prognosis, diagnosis and research methodology.

Our criterion for deciding that a review is ‘systematic’ is intentionally inclusive compared to available definitions ^10, 22, 23^. This is because we are explicitly interested in identifying systematic reviews with a range of methods, and not only those meeting a minimum standard of methods or reporting.

Our focus is on systematic reviews of public health and health systems interventions. Reviews in these areas are likely to feature diversity in included populations and settings, as well as intervention complexity ^24^. They are likely to include a range of study designs in addition to randomised trials, which in turn creates diversity in the effect measures used. Systematic reviews of public health and health systems interventions are more likely than other reviews to use synthesis methods other than meta-analysis ^9, 10^.

### Sample size

For reasons of feasibility, we will restrict the number of included reviews to 100. A sample of this size will allow us to estimate the proportion of reviews that use, for example, a particular synthesis or presentation method to within a maximum margin of error of 10%. This assumes a prevalence of 50%, but for a smaller or larger prevalence, the margin of error will be smaller. We anticipate that the proportion of reviews included in our sample that contain no meta-analyses will be approximately 50% ^9^.

### Search strategy

Records of all the systematic reviews published during 2018 will be obtained from two databases of systematic reviews: *Health Systems Evidence* and *Health Evidence* (see Table 2). These databases index systematic reviews of public health and health systems interventions, respectively.

**Table 2.** Source databases for cross-sectional sample of systematic reviews.

Database	Content coverage	Search strategy
Health Evidence www.healthevidence.org	Systematic reviews evaluating the effectiveness and cost- effectiveness of public health interventions ^26^	All records published in 2018 obtained
Health Systems Evidence www.healthsystemsevidence.org	Syntheses of research evidence about governance, financial and delivery arrangements within health systems, implementation strategies that can support change in health systems ^27^	Limits: Type = systematic review of effects Date range = 2018–2018

Description of Health Evidence and Health Systems Evidence database content, and limits used to obtain cross-sectional sample of systematic reviews for this study.

Some reviews identified by the search may have final citations outside 2018, for example arising from the difference between the date of online first publication and final publication in an issue of the journal, or the time lag between publication and indexing in a database. In these cases, the reference information will be updated to reflect the final citation, but reviews will not be excluded.

### Study selection

The records of systematic reviews retrieved from the two databases will initially be stored in Endnote and duplicate records removed. The selection and data extraction processes will then proceed using EPPI-Reviewer ^25^. Reviews will be randomly selected from this larger set using EPPI-Reviewer’s random selection function, and screened for eligibility until our target sample of 100 is met.

Records will be independently screened by two authors (MC and one of SB or JM) based on the title and abstract, and any clearly ineligible records excluded. The full text of potentially eligible SRs will then be retrieved and assessed independently against the eligibility criteria by one author (MC). A second author (either SB or JM) will assess the full text of a sample of 20% of records. At each stage, we will resolve any disagreements by consensus, and consult a third author if consensus is not possible.

For each included systematic review, any protocol or registration record referred to in the review will be retrieved. In addition, protocols will be retrieved for any systematic reviews published in the Cochrane or Campbell Libraries, as they are a requirement of publication in these journals.

### Data extraction and management

We will develop a data extraction form drawing on a previous methodological study that has examined synthesis and presentation methods used in systematic reviews ^21^, as well as frameworks and methods outlined in relevant guidance ^2– 4^.

We will collect data relating to the review characteristics, PICO characteristics used to group studies for each synthesis, and the synthesis methods used. Examples of data to be collected are presented in Table 1. The complete draft data dictionary is available online as *Extended data* ^19^. Both explicit methods described in the review and implicit methods observed in textual descriptions, tables and figures will be coded. Both planned and implemented methods will be collected where these differ.

In seeking to map current practice, we note that terms such as ‘narrative synthesis’ can be applied to a wide range of approaches, and will seek to identify specific components in our included reviews rather than relying on broad descriptive terms. We will collect:

- Sources of guidance referred to in the text.
- Methods of summary and synthesis explicitly specified in the Methods section.
- Methods of summary and synthesis used in practice (whether named or used implicitly in the text).
- Specific elements that may appear within a text-based summary approach, such as the use of consistent effect measures across studies, the use of non-parametric summary statistics such as ranges, various methods of vote counting, and the use of PICO groupings to structure text or tables.
- Explicit statements by the authors that they have been unable to implement planned PICO groupings or synthesis methods, their stated reasons for this, and what changes they made to their methods in response.

One author (MC) will extract data from all included reviews, and a second author (either SB or JM) will extract data independently on a sample of 20% of the included reviews (including those with and without meta-analysis). We will pilot test the data extraction form and coding guidance on five reviews to ensure we capture all items, to refine the items and guidance when we uncover ambiguity or a lack of clarity, and to achieve a shared understanding of the form. This will be achieved using an iterative process, where we discuss discrepancies and ambiguities as extraction is completed on each review, and revise the data extraction form and coding guidance in response to these discussions. Duplicate data extraction on the selected sample will then proceed, and agreement will be assessed at the end of this phase. For any data items in which a high degree of inconsistency is observed, duplicate data extraction will be undertaken for a further random sample of reviews. During the final, single data extraction phase, any uncertainties arising will be discussed with three authors (MC, SB, JM) and consensus reached.

We will limit our data collection to information contained in the published report(s) of the SR, including protocols and registry records, and will not contact authors to obtain additional information.

### Analysis

We will calculate descriptive summary statistics to characterise the extent to which authors specify their PICO for synthesis, and the synthesis and presentation methods. For example, the percentage of reviews where intervention groups are listed by name, are defined in enough detail for replication, and have an explicit role in the planned synthesis. Similarly, these percentages will be calculated for the other PICO elements.

For dichotomous or categorical data, we will calculate percentages and frequencies. For continuous or count data, we will calculate the means (with standard deviations) and medians (with interquartile ranges). We will examine whether approaches used to group the PICO for each synthesis are associated with the type of synthesis method by calculating differences in percentages between groups with 95% confidence intervals. Data will be tabulated and summarised in figures. Analyses will be undertaken using STATA ^28^.

### Dissemination

The findings of the research outlined in this protocol will be published. Associated datasets, data collection forms and analyses not included in any publication will be made publicly available via an online repository.

### Study status

At submission of this protocol, the search had been conducted and screening of abstracts completed. Full text screening and piloting of the data extraction form was in progress.

## Discussion

In this review, we will examine the methods choices for two intertwined elements of synthesis in systematic reviews. Namely, the approaches used to define and group PICO characteristics, and the types of synthesis methods other than meta-analysis. The results from our review will provide a snapshot of these practices, and highlight where improvements may be required in the application and reporting of the methods. Further, the study will provide a baseline assessment prior to release of recent guidance published in the *Cochrane Handbook of Systematic Reviews of Interventions* ^2– 4^, against which future assessments can be compared.

There are several strengths to our study. Our sample of systematic reviews is likely to be representative of public health and health systems intervention reviews because the source databases from which we will select our sample, and our inclusion criteria, place no restrictions on the intervention type or other features of the systematic reviews (e.g. the type of included study designs). A further strength is that our data extraction items are based on pre-existing frameworks to classify both the PICO groupings and methods of summary and synthesis. This will ensure that we are capturing specific methods and enhance the consistency of our data extraction.

There are some possible limitations in our proposed methods. For some items, the sample size may not be large enough to yield precise estimates of the percentage of systematic reviews that use particular methods. In addition, we will not undertake independent full text screening and data extraction of all studies by two authors, leaving some risk that data will be missed or misclassified. However, the review team has extensive experience in systematic reviews of public health and health services interventions, having written guidance for, co-authored, and edited many such reviews. While this will not mitigate missing information in the papers, it will help with making judgments required in the data extraction. Given that the aim of our study is to gain a broad understanding of current practice, we think this is unlikely to have an important impact on conclusions.

When complete, the findings of this study will be published and communicated at conferences, in addition to dissemination through international networks of researchers and authors of methodological guidance in the field of systematic reviews.

Authors of systematic reviews face challenges in the organisation and analysis of data, including the complexity of grouping studies for comparison, and synthesis methods when meta-analysis is not available. This protocol outlines the methods for a cross-sectional study that aims to examine the approaches used to define and group PICO characteristics, and the types of synthesis methods other than meta-analysis in a sample of systematic reviews of public health and health services interventions.

## Data availability

### Underlying data

No underlying data are associated with this article.

### Extended data

Figshare (Monash University repository, known as Bridges): Draft data dictionary for cross-sectional study of current practice in systematic reviews including the ‘PICO for each synthesis’ and methods other than meta-analysis. https://doi.org/10.26180/5edb178961d68 ^19^.

### Reporting guidelines

Figshare (Monash University repository, known as Bridges): PRISMA-P reporting checklist for protocol of cross-sectional study of current practice in systematic reviews including the ‘PICO for each synthesis’ and methods other than meta-analysis. https://doi.org/10.26180/5edb35183074f ^29^.

Data are available under the terms of the Creative Commons Attribution 4.0 International license (CC-BY 4.0).
