# Supplementary material for: The use of ‘PICO for synthesis’ and methods for synthesis without meta-analysis: protocol for a survey of current practice in systematic reviews of health interventions
Source: F1000Res. 2021 Jan 29;9:678. Originally published 2020 Jul 6. [Version 2] doi: 10.12688/f1000research.24469.2 (PMC7919603; doi:10.12688/f1000research.24469.2)
Supplement: Peer review history

# Peer review: Reviewer response for version 2

**DOI:** 10.5256/f1000research.54074.r78521

**Published:** 2021-02-26

**Authors:** Paul Whaley

**Affiliations:** 1 Lancaster Environment Centre, Lancaster University, Lancaster, UK; 2 Evidence-based Toxicology Collaboration, Johns Hopkins University Bloomberg School of Public Health, Baltimore, MD, USA

My responses to the authors' revisions, based on my numbered comments on their manuscript, are below. I am completely satisfied with the authors' responses and therefore pleased to "approve" it.

 Overall, I want to say that I very much enjoyed reviewing this protocol and learned a good deal from the authors' patient and thoughtful responses to my comments. Thank you. This is also my first open peer review. I found it constructive and enjoyable, and appreciate the transparency.

 I wish the authors all the best with their study and look forward to reading the final manuscript. Good luck!

 ---

1. The revised title is clear, no further comments.
2. The abstract reads very well, no further comments.
3. Good response, no further comments.
4. Good discussion of this point, no further comments.
5. No further comment.
6. This is a very thoughtful and clear response, thank you - no further comments.
7. The changes are clear, no further comment.
8. My apologies for missing the link to the "Extended Data". The authors are correct that this is exactly what I was looking for. The changes to make this information more prominent are welcome. No further comment.
9. I thank the authors for their patient and thorough response, no further comment.
10. The authors make a strong case for single reviewer extraction being appropriate in this study and I am convinced they have introduced appropriate safeguards to ensure sufficiently accurate extraction, no further comment.
11. The authors present a good argument for not conducting a more detailed piloting process. My concern arose partly from my missing the extended data from the original manuscript, which has been addressed, and partly from my experience as an editor working with relatively inexperienced review teams. For the latter, piloting during protocol development tends to be really important as it throws up important issues in the validity and application of the planned methods. For experienced teams using familiar methods, these concerns would diminish, so I am satisfied with the authors' response. No further comment.

Is the study design appropriate for the research question?

Partly

Is the rationale for, and objectives of, the study clearly described?

Partly

Are sufficient details of the methods provided to allow replication by others?

Partly

Are the datasets clearly presented in a useable and accessible format?

Not applicable

Reviewer Expertise:

Systematic review methods in environmental health research.

I confirm that I have read this submission and believe that I have an appropriate level of expertise to confirm that it is of an acceptable scientific standard.

# Peer review: Reviewer response for version 2

**DOI:** 10.5256/f1000research.54074.r78522

**Published:** 2021-02-11

**Authors:** Livia Puljak

**Affiliations:** 1 Center for Evidence-Based Medicine and Health Care, Catholic University of Croatia, Zagreb, Croatia

The authors have revised the manuscript adequately.

Is the study design appropriate for the research question?

Yes

Is the rationale for, and objectives of, the study clearly described?

Yes

Are sufficient details of the methods provided to allow replication by others?

Yes

Are the datasets clearly presented in a useable and accessible format?

Yes

Reviewer Expertise:

Research methodology, evidence synthesis, clinical epidemiology

I confirm that I have read this submission and believe that I have an appropriate level of expertise to confirm that it is of an acceptable scientific standard.

# Peer review: Reviewer response for version 1

**DOI:** 10.5256/f1000research.26991.r73015

**Published:** 2020-11-05

**Authors:** Paul Whaley

**Affiliations:** 1 Lancaster Environment Centre, Lancaster University, Lancaster, UK; 2 Evidence-based Toxicology Collaboration, Johns Hopkins University Bloomberg School of Public Health, Baltimore, MD, USA

**General comments**

 The authors present a protocol for a planned systematic review of meta-analysis and "other" methods for synthesising evidence in systematic reviews of public health and health systems interventions. It is definitely a novel, interesting, and worthwhile project of potentially very high value - I find myself in full agreement with the authors that "other" methods are underutilised and underdeveloped in SRs and a survey of current practices will provide valuable evidence for improvements in this area.

 However, I am not convinced that the proposed approach is the finished article. Besides some relatively trivial issues with the clarity of the objectives, I am particularly concerned about the lack of detail on how the authors are going to classify the methods employed in the included SRs. There is no comprehensive code book presented, so it is not possible to judge if the authors are gathering information which is going to optimally feed their intended analysis. The authors also discuss how various elements of the methodology will be piloted - but since this will resolve the issues with the protocol, I would strongly advocate that piloting being conducted now, and the protocol revised on the basis of that. I would definitely like to see a piloted code-book presented as part of the protocol.

 I think this is very promising work, just incomplete. It is also very challenging to communicate some of the issues as written feedback. I hope what I write below makes sense and is fair (or at least is constructive). If I over-explain in places, it is not because I am assuming the authors to be ignorant of their own subject matter but am attempting to provide context for a reader of the published peer-review. Since this review is not anonymous, I am sure the authors can track down my contact details easily enough. I would be more than happy to discuss my comments over the phone if they had any questions.

**Specific comments**

1. I am not completely sure if the title clearly and succinctly captures exactly what the review is about. For example, I see why the authors call it a "cross-sectional study", but it seems an odd occasion on which to use the term. Would it be plainer to call it something like: "A survey of current evidence synthesis practices in quantitative systematic reviews of public health and health systems interventions: study protocol" (I'm sure the authors can improve on this!).
2. The abstract is quite difficult to follow - I had to read the paper in order to really grasp what the abstract was about. The authors might want to reconsider how they are summarising their research, maybe focusing on how PICOs structure SRs and syntheses in the introduction. They should clearly state the objective (it's too compressed at the moment). The Methods section gives a lot of relatively trivial information about the extraction strategy, yet only has once sentence for the difficult and interesting part, which is the methodology for comparing approaches (what does it mean by their intent to "compare approaches" here? The summary seems too compressed.).
3. I wonder if the concept of "synthesis" is clearly enough introduced, or if it could be more directly introduced and defined at the outset. Currently, the concept is introduced after "however" in sentence 3 of para 1, contextualised by the claim of synthesis methods as often being narrowly considered. This seems back-to-front - would it be better to state what "synthesis" means, qualify this as being broader than some understandings, and then introduce the role of the PICO in structuring evidence synthesis (whether narrative or quantitative).
4. I am not sure about the terminology in splitting synthesis into meta-analysis vs. "other synthesis methods". There is a rich history of development and analysis of "other" methods, which I am sure the authors are aware of but they could perhaps use more fully. Often, the difference is defined as quantitative vs. either non-quantitative methods, narrative synthesis methods, or synthesis without meta-analysis ("SWIM"). This feels like contextual information that would be useful to the reader (given the author's assumption that there is a lack of awareness of this in the discipline) and might result in a choice of phrasing which better reflects established conventions.
5. This is a more trivial point. The authors state "Recent guidance published in the Cochrane Handbook for Systematic Reviews of Interventions has outlined proposed options in these two areas" - it would be helpful if these other areas were briefly indicated.
6. I am not sure if the discussion of reasons for not conducting meta-analysis is sufficiently nuanced. Certainly, lack of numeric data would challenge meta-analysis, but is a common issue not also excess heterogeneity between study designs? (Maybe this is less important for clinical SRs than it is in my field of environmental health.) Either way, in such cases narrative or SWIM methods come to the fore. They are complex, and I worry they are not sufficiently acknowledged in the rather broad statement that various synthesis methods such as harvest plots, etc. "may be preferable to textual description of the results in which there is a risk that authors may privilege the results of some studies over others without appropriate justification, possibly introducing bias". Echoing an earlier comment, I feel that this skates over some quite well-developed narrative or SWIM methodology which seeks to counter these concerns when providing textual summarisation. Introducing more of this theory I think is important for the protocol, coding the included SRs, and interpreting the methods in those SRs. (Note I use the term “narrative” in the sense of synthesis without quantitative methods, not in the sense of a narrative vs. systematic review.)
7. I wonder if it is worth the authors clarifying up-front that this is a survey of SRs of quantitative data, just to be clear that qualitative research is not the target of investigation. I think I only resolved this at the point of reading the eligibility criteria. Likewise the focus on public health and health systems interventions.
8. I am not clear as to how are the authors going to code and classify "other" methods? The authors have not provided a code book, so the utility and validity of their coding methodology cannot be evaluated. It seems like a potentially complex challenge, particularly if studies do not define their synthesis methods (it may be impossible for them to do so if there is no agreed way of categorising PICO-based synthesis approaches). The code definitions and criteria should be defined as far as possible in advance. I also note that in Table 2 "Examples of data collection items" that for summary and synthesis methods that the examples are almost exclusively related to quantitative methods and few examples of SWIM methods are given. This suggests to me that more planning around classifying these "other" methods is needed, to anticipate what they are and how they are accommodated in the analysis.
9. In terms of providing a code book, as handling editor, I gave the authors of this protocol a similarly hard time as I am giving the present authors. Table A3 may be instructive. https://doi.org/10.1016/j.envint.2020.105826. ^1^
10. I am also unclear as to how the concept of "PICO for each synthesis" is operationalised in the data extraction methods and the synthesis approach the authors will themselves be using. Introducing this concept seems a central concern of the introduction, yet by the methods section, it seems to have faded into the background. How is this concept going to structure the extraction and analysis, such that after conducting this survey we know how authors of SRs are using PICO statements in developing the synthesis components of their SRs?
11. I am not convinced that having just one person coding such complex data is sufficient. Coding decisions are likely to be difficult and require discussion. The authors seem to acknowledge this by requiring that 20% of extraction and coding be double-checked, but they provide no plan for what to do if that double-checking finds inconsistency. Is 80% agreement enough? Will the second extractor be trained to the same level as the first? If not, what does disagreement between the primary and secondary reviewer even mean? If agreement is low, do they intend to revise the coding criteria or seek to resolve disagreement? Will they double-check everything to ensure consistency across the full set of extracted data? I suspect it would be simpler, if more time-consuming, to train two extractors to an equal degree, code in parallel, then discuss the results to achieve considered consistency.
12. In terms of piloting, I would be much more comfortable if this was conducted and reported as part of the protocol development process. Since piloting is part of planning, I am not personally of the view that it is sufficient to state in a protocol that something will be piloted. Doing the piloting now would also help answer quite a few of the questions I have above.

Is the study design appropriate for the research question?

Partly

Is the rationale for, and objectives of, the study clearly described?

Partly

Are sufficient details of the methods provided to allow replication by others?

Partly

Are the datasets clearly presented in a useable and accessible format?

Not applicable

Reviewer Expertise:

Systematic review methods in environmental health research.

I confirm that I have read this submission and believe that I have an appropriate level of expertise to state that I do not consider it to be of an acceptable scientific standard, for reasons outlined above.

## References

[1] : Urban policy interventions to reduce traffic emissions and traffic-related air pollution: Protocol for a systematic evidence map. *Environ Int*.2020;142: 10.1016/j.envint.2020.105826 105826 10.1016/j.envint.2020.105826 32505921

# Response

**Published:** 2021-01-19

**Authors:** Miranda Cumpston

**Affiliations:** Monash University, Australia

We thank the reviewer for their constructive feedback and suggestions. We have found the reviewer’s perspective particularly valuable for understanding where more contextual background and methods detail is needed and have revised the text to address the concerns raised. Our response to each comment is presented below.

**General comments**

*The authors present a protocol for a planned systematic review of meta-analysis and "other" methods for synthesising evidence in systematic reviews of public health and health systems interventions. It is definitely a novel, interesting, and worthwhile project of potentially very high value - I find myself in full agreement with the authors that "other" methods are underutilised and underdeveloped in SRs and a survey of current practices will provide valuable evidence for improvements in this area.*

Response: We thank the reviewer for these comments.

**Specific comments**

*1. I am not completely sure if the title clearly and succinctly captures exactly what the review is about. For example, I see why the authors call it a "cross-sectional study", but it seems an odd occasion on which to use the term. Would it be plainer to call it something like: "A survey of current evidence synthesis practices in quantitative systematic reviews of public health and health systems interventions: study protocol" (I'm sure the authors can improve on this!).*

Response: We used the term ‘cross-sectional’ based on the title of other studies that have also used survey sampling methodology to sample the reviews (e.g. Page, et al. ^1^). However, we agree with the reviewer’s suggestion that ‘survey’ more accurately captures the study design. In addition, we have revised the title to be plainer, while still capturing the specific systematic review practices to be examined.

Changes made:

- The revised title is: “The use of ‘PICO for synthesis’ and methods for synthesis without meta-analysis: protocol for a survey of current practice in systematic reviews of health interventions”

*2. The abstract is quite difficult to follow - I had to read the paper in order to really grasp what the abstract was about. The authors might want to reconsider how they are summarising their research, maybe focusing on how PICOs structure SRs and syntheses in the introduction. They should clearly state the objective (it's too compressed at the moment). The Methods section gives a lot of relatively trivial information about the extraction strategy, yet only has once sentence for the difficult and interesting part, which is the methodology for comparing approaches (what does it mean by their intent to "compare approaches" here? The summary seems too compressed.).*

Response: We have revised the Abstract to describe our areas of interest more specifically. We have increased the level of detail in the Objectives and Methods, while acknowledging the constraint of the Abstract word limit.

*3. I wonder if the concept of "synthesis" is clearly enough introduced, or if it could be more directly introduced and defined at the outset. Currently, the concept is introduced after "however" in sentence 3 of para 1, contextualised by the claim of synthesis methods as often being narrowly considered. This seems back-to-front - would it be better to state what "synthesis" means, qualify this as being broader than some understandings, and then introduce the role of the PICO in structuring evidence synthesis (whether narrative or quantitative).*

Response: Reversing the order seemed reasonable, but after making this change, we realised that it did not work well. Synthesis is typically used to refer to the meta-analysis in reviews of health interventions, whereas the broader concept of synthesis that we use in this study is new, so it seemed best to retain an order in which we build from the status quo to the newer concept that is central to our study.

*4. I am not sure about the terminology in splitting synthesis into meta-analysis vs. "other synthesis methods". There is a rich history of development and analysis of "other" methods, which I am sure the authors are aware of but they could perhaps use more fully. Often, the difference is defined as quantitative vs. either non-quantitative methods, narrative synthesis methods, or synthesis without meta-analysis ("SWIM"). This feels like contextual information that would be useful to the reader (given the author's assumption that there is a lack of awareness of this in the discipline) and might result in a choice of phrasing which better reflects established conventions.*

Response: The split between meta-analysis and ‘other methods’ mirrors that in our work in the *Cochrane Handbook for Systematic Reviews of Interventions*, ^2^ but we now see that ‘other methods’ is too vague in this context. We have revised the heading to be more explanatory (see list of changes below).

 Terminology is something that we have debated at length with our co-authors as contributors to the Cochrane Handbook ^2-6^ and the SWiM reporting guidance. ^7^ We agree that it is important to follow convention, but in our view, there is a need to increase conceptual clarity in this area through clearer delineation of the steps and methods captured under the umbrella of ‘narrative synthesis’ (and synonyms such as ‘non-quantitative’ and SWiM). Used correctly, these terms are broader than our concept of ‘other synthesis methods’ where we refer to the methods used to *present and aggregate quantitative results* from two or more studies (for clarification of scope, see response to Comment 8). For this reason, we don’t use ‘narrative synthesis’ (or synonyms) for these methods, but have made revisions to address these conceptual issues.

Changes made:

- In the paragraph starting “Recent guidance …” (Introduction, para 3) we have added and referenced the following “Reporting guidance for ‘synthesis without meta-analysis’ (SWiM) has also been published …” ^7^
- At the end of the section on ‘PICO for synthesis’, we have added the following paragraph: “The PICO for synthesis also provides a framework for examining similarities and differences in the characteristics of studies contributing to each analysis, facilitating qualitative synthesis of characteristics needed to interpret results. This qualitative synthesis is a particularly import feature of reviews where there is diversity in study characteristics that may explain findings (e.g. intervention complexity, different study designs). ^6^ Such diversity sometimes triggers a decision not to use meta-analysis, and instead adopt alternative methods to synthesise and present findings. In these circumstances, it is common for authors to refer to their synthesis methods as ‘narrative’, ^8^ reflecting the integration of the synthesis of quantitative results from studies with the qualitative synthesis of study characteristics. In this study, we distinguish between these elements and, in the section that follows, focus on the methods used to combine quantitative data on intervention effects using a statistical technique and to present the results of these analyses.”
- Changed section heading in Introduction from ‘Other synthesis methods’ to ‘Synthesising and presenting findings without meta-analysis’. Note that we include the term ‘presenting’ to encompass structured summary of individual study results and visual presentation of data.

*5. This is a more trivial point. The authors state "Recent guidance published in the Cochrane Handbook for Systematic Reviews of Interventions has outlined proposed options in these two areas" - it would be helpful if these other areas were briefly indicated.*

Changes made:

- “Recent guidance published in the *Cochrane Handbook for Systematic Reviews of Interventions* ^2-4 ^has outlined proposed methods for specifying the PICO for each synthesis and a range of other synthesis methods.”

*6. I am not sure if the discussion of reasons for not conducting meta-analysis is sufficiently nuanced. Certainly, lack of numeric data would challenge meta-analysis, but is a common issue not also excess heterogeneity between study designs? (Maybe this is less important for clinical SRs than it is in my field of environmental health.) Either way, in such cases narrative or SWIM methods come to the fore. They are complex, and I worry they are not sufficiently acknowledged in the rather broad statement that various synthesis methods such as harvest plots, etc. "may be preferable to textual description of the results in which there is a risk that authors may privilege the results of some studies over others without appropriate justification, possibly introducing bias". Echoing an earlier comment, I feel that this skates over some quite well-developed narrative or SWIM methodology which seeks to counter these concerns when providing textual summarisation. Introducing more of this theory I think is important for the protocol, coding the included SRs, and interpreting the methods in those SRs. (Note I use the term “narrative” in the sense of synthesis without quantitative methods, not in the sense of a narrative vs. systematic review.)*

Response: Regarding the point about diversity (heterogeneity), we agree that this is a common reason given for not using meta-analysis, so we have addressed this in the manuscript (see list of changes below).

 Regarding consideration of narrative/SWiM methods, *a*s the reviewer notes, this issue relates closely to that covered in our response to Comment 4, where we consider the scope of narrative synthesis in relation to our project. We agree that there is benefit in expanding on these conceptual issues, as done in the new paragraph (see Comment 4). We provide further clarification of our perspective below.

 It is not our intention to minimise the complexity of methods encompassed by the concept of ‘narrative synthesis’. Instead, we believe that the components and process of narrative synthesis need to be disentangled to provide clear guidance for review authors on how to plan, conduct and report their methods. Following the lead of authors such as Melendez Torres, ^9^ and our own work on the Cochrane Handbook ^2-5^ and SWiM, ^7^ we see three main components commonly conceived as part of a narrative synthesis:

1. the planning work done to decide how studies will be grouped to address the review questions (the PICO for each synthesis),
2. the qualitative analysis of study characteristics (PICO/PECO and study design features) done to prepare for synthesis, interpret and explain the quantitative synthesis findings, and
3. the methods used to present and synthesise quantitative results from studies.

Our study will examine (1) and (3) of the above. It is not within the scope of this study to examine (2) or the process by which authors integrate the findings from (2) and (3). Although these latter elements are common in reviews reporting ‘narrative synthesis’, in our view they are essential features of any review where there is diversity (and complexity) of study characteristics that must be considered in order to identify and explain variation in effects across studies. While this may be described as the ‘narrative’ synthesis that ‘tells the story’ of the evidence, it can be done irrespective of whether meta-analysis is used or not.

Changes made:

- Addition of new paragraph addressing the concept of narrative synthesis and indicating that diversity of study characteristics may be a trigger for not using meta-analysis (as per comment 4) (‘PICO for each synthesis, final para).
- Added the following to address diversity: “Diversity of study characteristics and the presence of statistical heterogeneity are other reasons given for not meta-analysing, but these are more contentious. The first brings into question whether any synthesis is appropriate, the second may be addressed by using extensions to meta-analysis (e.g. meta-regression, prediction intervals) that attempt to explain or encompass heterogeneity.” (‘Synthesising and presenting findings without meta-analysis’, para 1)
- Edited the sentence that reads ‘…textual description of the results …’ to ‘Nevertheless, structured summary or synthesis approaches may be preferable to simply *presenting an unstructured inventory of study-level results*…’. We made this change to avoid any misunderstanding that by ‘textual description’ we are referring to narrative synthesis. (‘Synthesising and presenting findings without meta-analysis’, para 3)
- Amended our text to distinguish between structured summaries of the results of individual studies, and more unstructured summaries that are common in reviews.

*7. I wonder if it is worth the authors clarifying up-front that this is a survey of SRs of quantitative data, just to be clear that qualitative research is not the target of investigation. I think I only resolved this at the point of reading the eligibility criteria. Likewise the focus on public health and health systems interventions.*

Response: We have revised the text throughout to specify our focus on reviews of quantitative data and public health and services interventions, including the Abstract and Objectives.

*8. I am not clear as to how are the authors going to code and classify "other" methods? The authors have not provided a code book, so the utility and validity of their coding methodology cannot be evaluated. It seems like a potentially complex challenge, particularly if studies do not define their synthesis methods (it may be impossible for them to do so if there is no agreed way of categorising PICO-based synthesis approaches). The code definitions and criteria should be defined as far as possible in advance. I also note that in Table 2 "Examples of data collection items" that for summary and synthesis methods that the examples are almost exclusively related to quantitative methods and few examples of SWIM methods are given. This suggests to me that more planning around classifying these "other" methods is needed, to anticipate what they are and how they are accommodated in the analysis. In terms of providing a code book, as handling editor, I gave the authors of this protocol a similarly hard time as I am giving the present authors. Table A3 may be instructive. https://doi.org/10.1016/j.envint.2020.105826. 1*

Response: In addition to the example data collection items presented in the table, we provided a link to our draft data dictionary as Extended Data in the original version of this paper (‘Data extraction and management’ section and Data Availability statement, see https://doi.org/10.26180/5edb178961d68 ). The data dictionary provides the items, their response options, and a description of the items along with some guidance on coding. The dictionary covers both the data items relevant to the PICO for each synthesis (sections 2-6) and synthesis, summary and presentation methods (section 7). We believe the information provided in our data dictionary is as comprehensive as the example suggested by the reviewer.

 As noted in our response to Comments 4 and 6, our focus is on a subset of methods considered within the broader concept of narrative synthesis (or SWiM). Specifically, the planning of groups for synthesis (PICO for synthesis) and the methods used to present and synthesise quantitative results. The items we have included to capture the synthesis, summary and presentation methods are aligned with the methods we have outlined in Chapter 12 of the Cochrane Handbook, ‘Synthesizing and presenting findings using other methods’ ^2^ ( https://training.cochrane.org/handbook/current/chapter-12#section-12-2). We have successfully used these items in a previous study. ^10^

Changes made:

- Moved the reference to the data dictionary to the first paragraph of the ‘Data extraction and management’ section to increase its prominence.
- Added additional detail of the data items in Table 1 (note that the former Table 2 has been renumbered as it is now referenced earlier in the text), and added a reference to the full data dictionary in the table’s caption.
- Added the following paragraph to the ‘Data extraction and management’ section: “In seeking to map current practice, we note that terms such as ‘narrative synthesis’ can be applied to a wide range of approaches, and will seek to identify specific components in our included reviews rather than relying on broad descriptive terms. We will collect:

- Sources of guidance referred to in the text.
- Methods of summary and synthesis explicitly specified in the Methods section.
- Methods of summary and synthesis used in practice (whether named or used implicitly in the text).
- Specific elements that may appear within a text-based summary approach, such as the use of consistent effect measures across studies, the use of non-parametric summary statistics such as ranges, various methods of vote counting, and the use of PICO groupings to structure text or tables.
- Explicit statements by the authors that they have been unable to implement planned PICO groupings or synthesis methods, their stated reasons for this, and what changes they made to their methods in response.”

*9. I am also unclear as to how the concept of "PICO for each synthesis" is operationalised in the data extraction methods and the synthesis approach the authors will themselves be using. Introducing this concept seems a central concern of the introduction, yet by the methods section, it seems to have faded into the background. How is this concept going to structure the extraction and analysis, such that after conducting this survey we know how authors of SRs are using PICO statements in developing the synthesis components of their SRs?*

Response: Details of how the PICO for synthesis is operationalised are summarised in Table 1, with full details provided in the draft data dictionary. We will undertake a descriptive analysis to characterise the extent to which authors specify their PICO for synthesis, and the basis for their PICO. For example, the percentage of reviews where intervention groups are listed by name, are defined in enough detail for replication, and have an explicit role in the planned synthesis. Similarly, these percentages will be calculated for the other PICO elements.

 For each PICO element and study design, we capture any groupings identified, whether an explicit role in synthesis is reported, whether the groupings were specified at the level of detail required for replication, whether the specified groupings were used in practice, whether additional new groupings were used in practice that were not specified, and statements by the authors describing any change in the planned groupings.

 In addition, we describe the basis of the groupings (such as whether groupings are based on existing taxonomies, or whether they are based on e.g. disease categories, health equity characteristics, time of measurement, etc.) using categories drawn from the frameworks provided for each PICO element in Chapter 3, Section 3.2 of the *Cochrane Handbook for Systematic Reviews of Interventions* *^4^* (available free online at https://training.cochrane.org/handbook/current/chapter-03#section-3-2).

Changes made:

- Added additional detail of the data items collected to examine the PICO for each synthesis in Table 1 (previously Table 2).
- Added the following text to the analysis section “We will calculate descriptive summary statistics to characterise the extent to which authors specify their PICO for synthesis, and the synthesis and presentation methods. For example, the percentage of reviews where intervention groups are listed by name, are defined in enough detail for replication, and have an explicit role in the planned synthesis. Similarly, these percentages will be calculated for the other PICO elements.”

*10. I am not convinced that having just one person coding such complex data is sufficient. Coding decisions are likely to be difficult and require discussion. The authors seem to acknowledge this by requiring that 20% of extraction and coding be double-checked, but they provide no plan for what to do if that double-checking finds inconsistency. Is 80% agreement enough? Will the second extractor be trained to the same level as the first? If not, what does disagreement between the primary and secondary reviewer even mean? If agreement is low, do they intend to revise the coding criteria or seek to resolve disagreement? Will they double-check everything to ensure consistency across the full set of extracted data? I suspect it would be simpler, if more time-consuming, to train two extractors to an equal degree, code in parallel, then discuss the results to achieve considered consistency.*

Response: We recognise that a potential limitation of the review is that we will not undertake double data extraction of all reviews; this is due to limited resources. However, we are investing substantial time and effort in the piloting stage to refine the items and guidance, and achieve a shared understanding of the form (see below for the changes made in this regard). The team undertaking the data extraction have extensive experience in systematic reviews of public health and health services interventions, having written guidance for, co-authored, and edited many such reviews. While we appreciate the experience of the team does not mitigate missing information in the papers (a point which we note in the discussion of the revised protocol), it does help with making the judgments required in the data extraction. Finally, we believe that the consequences of some errors in the data extraction in a methodological review such as this are less serious than that of reviews examining the effects of interventions and environmental exposures, where misleading results arising from errors in the data extraction can impact policy decisions, patients’ health outcomes and quality of care.

Changes made:

- We have revised the ‘Data extraction and management’ section of the paper to provide more detail on our proposed approach: “One author (MC) will extract data from all included reviews, and a second author (either SB or JM) will extract data independently on a sample of 20% of the included reviews (including those with and without meta-analysis). We will pilot test the data extraction form and coding guidance on five reviews to ensure we capture all items, to refine the items and guidance when we uncover ambiguity or a lack of clarity, and to achieve a shared understanding of the form. This will be achieved using an iterative process, where we discuss discrepancies and ambiguities as extraction is completed on each review, and revise the data extraction form and coding guidance in response to these discussions. Duplicate data extraction on the selected sample will then proceed, and agreement will be assessed at the end of this phase. For any data items in which a high degree of inconsistency is observed, duplicate data extraction will be undertaken for a further random sample of reviews. During the final, single data extraction phase, any uncertainties arising will be discussed with three authors (MC, SB, JM) and consensus reached.” (para 3)
- We have added the following text to the Discussion: “However, the review team has extensive experience in systematic reviews of public health and health services interventions, having written guidance for, co-authored, and edited many such reviews. While this will not mitigate missing information in the papers, it will help with making judgments required in the data extraction. Given that the aim of our study is to gain a broad understanding of current practice, we think this is unlikely to have an important impact on conclusions.” (para 3)

*11. In terms of piloting, I would be much more comfortable if this was conducted and reported as part of the protocol development process. Since piloting is part of planning, I am not personally of the view that it is sufficient to state in a protocol that something will be piloted. Doing the piloting now would also help answer quite a few of the questions I have above.*

Response: We agree that piloting is an important aspect of a systematic review. Aspects of the data extraction for the present study (i.e. other synthesis methods items) were based on a previous study we undertook, where we had high concordance in coding across reviewers (including both experienced methodologists, and those with limited experience). We note that the current data extraction form is based on a previous study in the ‘Data extraction and management’ section.

 We note that the reviewer suggests that piloting should be conducted and reported as part of the protocol development process. We are not aware that there is an established convention for this. For example, it is not a requirement in Cochrane review protocols – which outline the methods for reviews evaluating the effects of healthcare interventions – to report a completed piloting process.

 Our protocol as submitted provides our planned methods at a point in time. We will report changes to those methods in our final report, including the finalised data dictionary arising from the piloting. We have used this approach in other methods reviews. ^11-13^ For example, in Arnup et al, ^11^ we undertook a review examining statistical methods used in cluster-randomised crossover trials. In a supplementary file to that paper, Table S1, we outlined changes in methods from protocol, and included the final data extraction form (which incorporated modifications from piloting).

**References**

 1. Page MJ, Shamseer L, Altman DG, Tetzlaff J, Sampson M, Tricco AC, et al. Epidemiology and Reporting Characteristics of Systematic Reviews of Biomedical Research: A Cross-Sectional Study. PLOS Medicine. 2016;13(5):e1002028.

 2. McKenzie J, Brennan S. Chapter 12: Synthesizing and presenting findings using other methods. In: Higgins J, Thomas J, Chandler J, Cumpston M, Li T, Page M, et al., editors. Cochrane Handbook for Systematic Reviews of Interventions. 2nd ed. Chichester (UK): John Wiley & Sons; 2019.

 3. McKenzie J, Brennan S, Ryan R, Thomson H, Johnston R. Chapter 9: Summarizing study characteristics and preparing for synthesis. . In: Higgins J, Thomas J, Chandler J, Cumpston M, Li T, Page M, et al., editors. Cochrane Handbook for Systematic Reviews of Interventions. 2nd ed. Chichester (UK): John Wiley & Sons; 2019.

 4. McKenzie J, Brennan S, Ryan R, Thomson H, Johnston R, Thomas J. Chapter 3: Defining the criteria for including studies and how they will be grouped for the synthesis. In: Higgins J, Thomas J, Chandler J, Cumpston M, Li T, Page M, et al., editors. Cochrane Handbook for Systematic Reviews of Interventions. 2nd ed. Chichester (UK): John Wiley & Sons; 2019.

 5. Thomas J, Kneale D, McKenzie J, Brennan S, Bhaumik S. Chapter 2: Determining the scope of the review and the questions it will address. In: Higgins J, Thomas J, Chandler J, Cumpston M, Li T, Page M, et al., editors. Cochrane Handbook for Systematic Reviews of Interventions. 2nd ed. Chichester (UK): John Wiley & Sons; 2019.

 6. Thomas J, Petticrew M, Noyes J, Chandler J, Rehfuess E, Tugwell P, et al. Chapter 17: Intervention complexity. In: Higgins J, Thomas J, Chandler J, Cumpston M, Li T, Page M, et al., editors. Cochrane Handbook for Systematic Reviews of Interventions. 2nd ed. Chichester (UK): John Wiley & Sons; 2019.

 7. Campbell M, McKenzie JE, Sowden A, Katikireddi SV, Brennan SE, Ellis S, et al. Synthesis without meta-analysis (SWiM) in systematic reviews: reporting guideline. BMJ. 2020;368:l6890.

 8. Campbell M, Katikireddi SV, Sowden A, Thomson H. Lack of transparency in reporting narrative synthesis of quantitative data: a methodological assessment of systematic reviews. J Clin Epidemiol. 2019;105:1-9.

 9. Melendez-Torres GJ, O'Mara-Eves A, Thomas J, Brunton G, Caird J, Petticrew M. Interpretive analysis of 85 systematic reviews suggests that narrative syntheses and meta-analyses are incommensurate in argumentation. Research Synthesis Methods. 2017;8(1):109-18.

 10. McKenzie J, Brennan S, Page M, Chau M, Kramer S, Bosch M. From summary to synthesis: a review of statistical synthesis and presentation methods used in complex reviews [poster].  Better Knowledge for Better Health | Un meilleur savoir pour une meilleure santé Abstracts of the 21st Cochrane Colloquium; 19-23 September 2013; Québec City, Canada: John Wiley & Sons; 2013.

 11. Arnup SJ, Forbes AB, Kahan BC, Morgan KE, McKenzie JE. Appropriate statistical methods were infrequently used in cluster-randomized crossover trials. J Clin Epidemiol. 2016;74:40-50.

 12. Page MJ, Forbes A, Chau M, Green SE, McKenzie JE. Investigation of bias in meta-analyses due to selective inclusion of trial effect estimates: empirical study. BMJ Open. 2016;6(4):e011863.

 13. Turner SL, Karahalios A, Forbes AB, Taljaard M, Grimshaw JM, Cheng AC, et al. Design characteristics and statistical methods used in interrupted time series studies evaluating public health interventions: a review. J Clin Epidemiol. 2020;122:1-11.

# Peer review: Reviewer response for version 1

**DOI:** 10.5256/f1000research.26991.r69547

**Published:** 2020-09-09

**Authors:** Livia Puljak

**Affiliations:** 1 Center for Evidence-Based Medicine and Health Care, Catholic University of Croatia, Zagreb, Croatia

The study proposed is interesting and relevant. The authors should be congratulated for this initiative. My only concern is the sample size. I am aware that the authors have indicated that 100 reviews are proposed to be analyzed due to feasibility reasons, but 100 reviews in this particular context appears like a very small sample to analyze heterogeneity of approaches that could be expected. So my concern is about generalizability of the results with such a small sample. It is unclear how many reviews will be found by the proposed search methods, and what percent of reviews will be analyzed when the sample of reviews is 100.

Is the study design appropriate for the research question?

Yes

Is the rationale for, and objectives of, the study clearly described?

Yes

Are sufficient details of the methods provided to allow replication by others?

Yes

Are the datasets clearly presented in a useable and accessible format?

Yes

Reviewer Expertise:

Research methodology, evidence synthesis, clinical epidemiology

I confirm that I have read this submission and believe that I have an appropriate level of expertise to confirm that it is of an acceptable scientific standard, however I have significant reservations, as outlined above.

# Response

**Published:** 2020-11-04

**Authors:** Miranda Cumpston

**Affiliations:** Monash University, Australia

We would like to thank Professor Puljak for her review of our study protocol.

 In the following, we respond to the two issues raised regarding sample size and generalisability. In this cross-sectional study we are using survey sampling methodology to sample the reviews (not aiming to locate and include all reviews meeting our eligibility criteria). Random (or probability) sampling allows valid estimates of population characteristics to be made (e.g. estimating the prevalence of reviews that use vote counting) along with calculation of uncertainty in those estimates [1]. The estimate of uncertainty (margin of error) is unchanged by the population size (total number of reviews), except in the circumstance where the fraction of reviews sampled is greater than approximately 5% of the population of reviews, in which case the margin of error will be appreciably less than we have specified in the protocol.

 We believe that the sample size of 100 reviews will be generally be sufficient such that the interpretation of the confidence limits will be consistent. For example, if we estimate the percentage of reviews using a particular synthesis method (e.g. vote counting based on the direction of effect) is 15%, the associated 95% confidence limits would be 8% to 22%, and our interpretation of these limits would lead to the same conclusion that the method was not commonly used. As another example, if the outcome was ‘reviewers fully specified the groups to be used in the synthesis’, and 50% of the reviewers were found to fully specify the groups, our interpretation would not differ at the limits of the confidence interval, which in this example would be from 40% to 60%. As noted in the protocol, the maximum margin of error will be 10%, which will occur for an estimated prevalence of 50%.

 In regard to generalisability (external validity), which we interpret as the extent to which our findings will be applicable to systematic reviews other than those examining public health or health systems interventions, we would be reluctant to generalise to other review types (e.g. prognostic reviews, diagnostic test accuracy reviews). However, importantly, the generalisability of study findings is not dependent on the sample size or statistical power, but on considering whether the reviews included in our study have characteristics that differ from another population of reviews to which the reader might wish to generalise the findings [2]. In reporting the findings of this study, we will ensure that the characteristics of the included studies are clearly summarised to facilitate such judgements by the reader.

 References:

 1. Kish, L., *Survey sampling*. 1965, New York: John Wiley & Sons, Inc.

 2. Kukull, W.A. and M. Ganguli, *Generalizability: the trees, the forest, and the low-hanging fruit.* Neurology, 2012. **78**(23): p. 1886-91.
